# Gabapentin‐Induced Hypoglycemia in a Patient With Pre‐Diabetes: A Case Report and Review of the Literature

**DOI:** 10.1002/ccr3.73324

**Published:** 2026-08-07

**Authors:** Reem Hussain Alamri, Abdullah Ali Alshamrani, Hesham E. Mawar, Suhaib Radi

**Affiliations:** ^1^ Department of Internal Medicine, Division of Endocrinology Ministry of the National Guard‐Health Affairs Jeddah Saudi Arabia; ^2^ King Abdullah International Medical Research Center Jeddah Saudi Arabia; ^3^ College of Medicine King Saud Bin Abdulaziz University for Health Sciences Jeddah Saudi Arabia

**Keywords:** adverse drug reaction, case report, gabapentin, hypoglycemia, pre‐diabetes

## Abstract

Gabapentin, a *γ*‐aminobutyric acid (GABA) analogue, is widely prescribed for neuropathic pain and partial seizures. While common adverse effects include somnolence and dizziness, hypoglycemia is rarely reported. A 76‐year‐old woman with hypertension, dyslipidemia, hypothyroidism, and pre‐diabetes (HbA1c 6%) developed recurrent symptomatic hypoglycemia shortly after starting gabapentin 300 mg once daily for neuropathic pain. Within two days, she experienced dizziness, sweating, and fatigue with capillary glucose readings as low as 2.4 mmol/L, confirmed on laboratory chemistry. Investigations revealed inappropriately elevated insulin and C‐peptide with negative ketones, consistent with a non‐ketotic hyperinsulinemic hypoglycemic state. Other causes, including insulinoma, factitious hypoglycemia, and adrenal insufficiency, were excluded. The Naranjo Adverse Drug Reaction Probability Scale score was 7, indicating a probable relationship between gabapentin and the hypoglycemia. Gabapentin was discontinued after three doses. The hypoglycemic episodes resolved within several days, and no further events occurred during hospital observation, a 72‐h fast, or continuous glucose monitoring at follow‐up. This case highlights gabapentin as a potential cause of clinically significant hypoglycemia, even in patients without overt diabetes. Clinicians should remain aware of this underrecognized adverse effect, especially in elderly patients.

## Key Clinical Message

Gabapentin can uncommonly trigger clinically significant hypoglycemia, even in patients with prediabetes and no renal impairment. Early recognition and a medication review are essential in cases of unexplained low blood glucose, as prompt discontinuation may prevent recurrent or severe hypoglycemic events.

## 1 Introduction

Gabapentin is an anticonvulsant and neuropathic pain medication that received Food and Drug Administration approval in 1993 and has been available in generic form since 2004. Although it is structurally related to *γ*‐aminobutyric acid (GABA), it exerts its therapeutic effects primarily through binding to the *α*2*δ* subunit of voltage‐gated calcium channels in the central nervous system [1]. Common adverse reactions include somnolence, dizziness, and sedation [1].

Blood glucose disturbances are listed among the possible adverse drug reactions (ADRs), but hypoglycemia remains an uncommon and underrecognized reaction. Only scattered reports describe this association, mainly in patients with diabetes, renal impairment, or in those on peritoneal dialysis [2, 3, 4]. This paper presents a case of recurrent hypoglycemia in a woman with prediabetes following the initiation of gabapentin.

## 2 Case Presentation

In April 2025, a 76‐year‐old woman presented with recurrent episodes of sweating, dizziness, and fatigue. Her past medical history included hypertension, dyslipidemia, hypothyroidism, and prediabetes (HbA1c 6.0%), for which she was taking metformin. She had no prior history of hypoglycemia or a history of ischemic heart disease. Her regular medications included metformin 500 mg twice daily, valsartan 160 mg twice daily, bisoprolol 5 mg once daily, levothyroxine 100 mcg once daily, and atorvastatin 10 mg once daily.

She had been recently been prescribed gabapentin 300 mg once daily at a private clinic for neuropathic pain, to be taken at 10:00 AM. On the second day of therapy, she developed recurrent episodes of symptomatic hypoglycemia approximately 16 h after drug intake, occurring around 2:00 AM. These episodes were documented by her caregiver using a home glucometer with readings ranging between 2.4 and 2.8 mmol/L (N: 3.9–7.8 mmol/L) mainly at night, while morning glucose values ranged between 6.4–7.6 mmol/L. On day 3, she sought medical attention as the episodes did not resolve. Upon presentation to the emergency department, her vital signs were within normal limits: blood pressure 132/78, heart rate 65 beats per minute, respiratory rate 20 breaths per minute, oxygen saturation 98% on room air, and temperature 36.7°C. Laboratory testing in the emergency department confirmed a plasma glucose of 2.4 mmol/L. Blood samples were sent for insulin, C‐peptide, and *β*‐hydroxybutyrate levels, and she was discharged from the emergency department. However, she continued to experience nocturnal hypoglycemia, with a lowest documented reading of 2.2 mmol/L.

## 3 Investigations

Laboratory investigations revealed normal liver enzymes and renal function (eGFR 91 mL/min/1.73 m^2^). Thyroid‐stimulating hormone, free T4, and morning cortisol levels were also within normal limits.

During a documented hypoglycemic episode, the plasma glucose was 2.4 mmol/L, with undetectable *β*‐hydroxybutyrate, and insulin and C‐peptide values of 2593 μU/mL (*N* < 26.1) and 3179 pmol/L (*N* < 200), respectively. Venous blood gas analysis showed a pH of 7.38, with normal pCO_2_, bicarbonate, and lactate levels. This pattern suggested a nonketotic hyperinsulinemic hypoglycemic state.

## 4 Management and Outcome

Gabapentin was discontinued after three doses, but hypoglycemia persisted for three additional days before gradually resolving. On day 6, the patient was admitted for a supervised 72‐h fasting test, which she completed without any symptomatic or documented hypoglycemia; plasma glucose fasting levels during the supervised 72‐h fasting test ranged from 7.7 to 10 mmol/L. At two‐ and four‐week outpatient follow‐ups, she remained asymptomatic, and continuous glucose monitoring demonstrated a stable glucose profile with no further hypoglycemic episodes.

## 5 Differential Diagnosis

The differential diagnosis for adult hypoglycemia includes:

- For ill or medicated patients: drug‐induced hypoglycemia, critical illness, hormone deficiency (adrenal insufficiency or hypothyroidism)
- For seemingly well patients: insulinoma, insulin autoimmune hypoglycemia, nesidioblastosis, and factitious hypoglycemia [5].

In the present case, the patient had no history of diabetes, and upon reviewing her medication, it was confirmed that no insulin, meglitinides, or sulfonylurea were prescribed to the patient. Other possible medications reported to cause hypoglycemia, including renin–angiotensin–aldosterone system inhibitors, non‐selective *β*‐blockers, metformin, and pentamidine, were also considered [5, 6]. Among these medications, the patient was taking metformin and valsartan. While valsartan‐induced hypoglycemia mostly happens in the context of valsartan/sacubitril, isolated case reports of valsartan monotherapy‐induced hypoglycemia exist in the literature [7]. However, three features argue against these agents as the primary cause: (1) both medications had been taken at stable unchanged doses for several years without any prior hypoglycemic episodes and without any recent dose adjustments or new clinical factors affecting their metabolism or clearance; (2) the biochemical pattern of markedly elevated endogenous insulin and C‐peptide is mechanistically consistent with gabapentin's insulinotropic effect, but not with valsartan or metformin [6, 7]; and (3) hypoglycemia resolved completely after gabapentin discontinuation alone, while both medications were continued unchanged throughout. A finding most difficult to reconcile with either agent being the primary driver. Nevertheless, a minor additive contribution cannot be entirely excluded. Critical illness–related hypoglycemia can occur in the setting of hepatic failure, renal failure, sepsis, starvation, or cardiac failure, where glucose utilization exceeds glucose intake, glycogenolysis, or gluconeogenesis [5, 8]. In addition, alcohol use and a deficiency of counter‐regulatory hormones, most notably cortisol, can impair glucose homeostasis and precipitate hypoglycemia [5, 9]. In the current case, there was no clinical or biochemical evidence of critical illness, hepatic or renal dysfunction, adrenal insufficiency, or alcohol use.

The laboratory findings in the patient demonstrate inappropriately elevated insulin levels with negative ketones, consistent with a nonketotic hyperinsulinaemic hypoglycaemia. Differential diagnosis for this biochemical pattern includes factitious hypoglycemia, insulinoma, insulin autoimmune hypoglycaemia, and nesidioblastosis [5].

Factitious hypoglycemia was considered highly unlikely, as the patient's biochemical profile demonstrated concurrent elevations of both insulin and C‐peptide, inconsistent with self administration of insulin, which typically suppresses endogenous C‐peptide leading to high insulin and low C‐peptide [10]. Additionally, the abrupt onset of hypoglycemia following the initiation of gabapentin, complete resolution after its discontinuation, and the absence of recurrent hypoglycemia during fasting or at follow‐up argue against insulinoma; furthermore, there were no clinical features suggestive of multiple endocrine neoplasia type 1. A Naranjo ADR probability score of 7 (Table 1) indicated that gabapentin was a probable causative agent [11].

**TABLE 1:** Naranjo Adverse Drug Reaction Probability Score [11].

Question	Yes	No	Do Not Know	Patient's score
1. Are there previous conclusive reports on this reaction?	1	0	0	1
2. Did the adverse event appear after the suspected drug was administered?	2	−1	0	2
3. Did the adverse reaction improve when the drug was discontinued or a specific antagonist was administered?	1	0	0	1
4. Did the adverse reaction reappear when the drug was readministered?	2	−1	0	0
5. Are there alternative causes (other than the drug) that could have caused the reaction?	−1	2	0	2
6. Did the reaction reappear when a placebo was given?	−1	1	0	0
7. Was the drug detected in blood (or other fluids) in concentrations known to be toxic?	1	0	0	0
8. Was the reaction more severe when the dose was increased, or less severe when the dose was decreased?	1	0	0	0
9. Did the patient have a similar reaction to the same or similar drugs in any previous exposure?	1	0	0	0
10. Was the adverse event confirmed by any objective evidence?	1	0	0	1
Total Score				7

*Note:* This probability scale is a validated 10‐item questionnaire used to determine the likelihood that a suspected drug caused an adverse reaction. The scale scores each question as Yes, No, or Do Not Know, with assigned values that can be positive, negative, or zero, depending on the question. Negative scores reflect responses that reduce the likelihood of an ADR [11]. Scoring: total score ≥ 9 = definite ADR; 5–8 = probable ADR; 1–4 = possible ADR; 0 = doubtful ADR [9].

## 6 Discussion

Gabapentin is widely prescribed for neuropathic pain with a well‐characterized ADR profile, which includes central nervous system effects and, less commonly, metabolic disturbances [1]. Hypoglycemia is not known as an ADR of gabapentin, and its occurrence is rarely reported, with approximately nine cases reported in the literature [2, 3, 4].

A review of previously reported cases (Table 2) highlights that gabapentin‐induced hypoglycemia has been observed across a wide age range (36–76 years), in both diabetic and nondiabetic individuals, at varying doses (300–1800 mg/day), and with different renal function status, it has also been reported in the setting of an overdose [12]. The resolution of hypoglycemia occurred in all patients with attempted dechallenge (reducing or stopping the medication), including this case. However, three of the eight cases in the literature did not attempt to dechallenge, did not require discontinuing the medication, and hypoglycemia did not recur [2, 3]. The present case is unique given the patient's advanced age, prediabetes, rapid onset of hypoglycemia after a low dose of gabapentin, and biochemical confirmation of hyperinsulinemia.

**TABLE 2:** Clinical characteristics of nine Gabapentin‐induced hypoglycemia compared to the present case.

Authors (Year)	Age/Sex	Diabetes status	Gabapentin dose (mg/day)	Renal function	Onset after initiation (days)	Dechallenge outcome (days to resolution)	Nadir blood glucose (mmol/L)	C‐peptide	Naranjo ADR score
Penelope et al. (2003) [2]	58 F	No	600	ESRD	30^a^	Positive^c^ (3 days)	1.9	Elevated	NR
Scholl et al. (2015) [3]	36 F	NR	1800	NR	30	4 positives^b^ (NR days), 2 not attempted	1.6	NR	Possible
	54 F	No	NR		21		1.3		Probable
	59 M	Yes	NR		NR		NR		Possible
	71 F	Yes	NR		NR		NR		Probable
	68 M	Yes	NR		NR		1.7		Possible
	60 M	Yes	NR		NR		3.9		Possible
Hayes et al. (2022) [4]	47 F	No	300	Normal	7	Not attempted	1.83	NR	Probable
Ghnaima et al. (2023) [12]	36 F	No	72,000^c^	Normal	< 1	NR	3.2	NR	NR
Present study	76 F	Pre‐diabetes	300	Normal	1	Positive^d^ (4 days)	2.2	Elevated	Probable

*Note:* Dechallenge outcome: positive, adverse event resolved after drug discontinuation or dose reduction; not attempted, dechallenge not attempted.

Abbreviations: ADR, adverse drug reaction; ESRD, end stage renal disease; F, female; M, male; NR, not reported.

^a^ Patient was taking the drug for 2 years but doubled the dose 1 month before hypoglycemia episode.

^b^ A single case of hypoglycemia of the 4 positives resolved after decreasing gabapentin dose and not stoppage.

^c^ This represents a case of gabapentin overdose.

^d^ Positive dechallenge with the discontinuation of the drug.

The mechanism by which gabapentin can induce hypoglycemia is not well‐established. However, there are two potential mechanisms that can be considered. The first is related to GABA receptor activation. GABA receptors are located in the cytoplasm, insulin granules, and microvesicles of pancreatic *β*‐cells [13]. It has two subtypes: GABA_A_ and GABA_B_. The activation of GABA_A_ leads to calcium influx, and therefore the release of insulin, whereas the activation of GABA_B_ leads to the inhibition of insulin release. Therefore, the theoretic gabapentin‐induced activation of GABA_A_ receptors may enhance insulin secretion, leading to hypoglycemia. However, this is thought to be unlikely, as the drug does not show receptor binding affinity in other body parts [1]. Another more probable mechanism involves voltage‐gated calcium channels. These channels are composed of *α*1, *β*, *α*2*δ*, and *γ* subunits. Gabapentin has a high affinity to bind specifically to the *α*2*δ*1 and *α*2*δ*2 isoforms [14]. While α2δ1 is predominantly expressed in neuronal tissue, α2δ2 subunits are present in the pancreas [15], and their activation may facilitate calcium entry into *β*‐cells, thereby promoting insulin secretion. The present case supports these mechanisms, as evidenced by the endogenous hyperinsulinemic biochemical pattern.

This case underscores the importance of monitoring for hypoglycemia in patients prescribed gabapentin. This includes patients with low doses of gabapentin and in patients without diabetes or renal impairment. Clinicians should be aware that hypoglycemia can occur within days of initiation and may present with nocturnal symptoms. Measurement of glucose, insulin, and C‐peptide is useful to confirm a hyperinsulinemic pattern; 72‐h fasting test is helpful for the exclusion of insulinoma, and discontinuation or dose reduction of gabapentin generally leads to resolution of hypoglycemia within days.

The scarcity of detailed case reports limits understanding of predisposing factors and the precise incidence of gabapentin‐induced hypoglycemia. Further studies are needed to identify high‐risk populations and provide clear guidance on monitoring. In addition, formal rechallenge with gabapentin was not performed in the present case due to ethical considerations given the severity of the hypoglycemic episodes and the patient's advanced age. While this could limit definitive ADR causality attribution, the Naranjo probability score of 7, combined with the clear temporal relationship between gabapentin initiation and hypoglycemia onset and its resolution upon discontinuation, supports a probable causal relationship.

## 7 Conclusion

Gabapentin may cause clinically significant hypoglycemia even in patients without significant comorbidities. Clinicians should maintain a high index of suspicion for drug‐induced hypoglycemia and consider medication review in patients with unexplained low glucose levels. This report adds to the literature by documenting the earliest onset reported to date in therapeutic doses, with laboratory‐confirmed non‐ketotic hyperinsulinemic hypoglycemia.

## Author Contributions

**Reem Hussain Alamri:** conceptualization, writing – original draft, project administration. **Abdullah Ali Alshamrani:** writing – review and editing, data curation. **Hesham E. Mawar:** writing – review and editing, writing – original draft. **Suhaib Radi:** writing – review and editing, supervision.

## Funding

The authors have nothing to report.

## Consent

Written informed consent was obtained from the patient for publication of this case report with all personal identifiers removed to ensure anonymity.

## Conflicts of Interest

The authors declare no conflicts of interest.

## Data Availability

The data that support the findings of this study are available on request from the corresponding author. The data are not publicly available due to privacy or ethical restrictions.
